# P2X7 receptors induce degranulation in human mast cells

**DOI:** 10.1007/s11302-016-9497-4

**Published:** 2016-02-24

**Authors:** Kathryn J. Wareham, Elizabeth P. Seward

**Affiliations:** University of Sheffield, Western Bank, Sheffield, S10 2TN UK

**Keywords:** P2X, Mast cells, ATP, Degranulation

## Abstract

Mast cells play important roles in host defence against pathogens, as well as being a key effector cell in diseases with an allergic basis such as asthma and an increasing list of other chronic inflammatory conditions. Mast cells initiate immune responses through the release of newly synthesised eicosanoids and the secretion of pre-formed mediators such as histamine which they store in specialised granules. Calcium plays a key role in regulating both the synthesis and secretion of mast-cell-derived mediators, with influx across the membrane, in particular, being necessary for degranulation. This raises the possibility that calcium influx through P2X receptors may lead to antigen-independent secretion of histamine and other granule-derived mediators from human mast cells. Here we show that activation of P2X7 receptors with both ATP and BzATP induces robust calcium rises in human mast cells and triggers their degranulation; both effects are blocked by the P2X7 antagonist AZ11645373, or the removal of calcium from the extracellular medium. Activation of P2X1 receptors with αβmeATP also induces calcium influx in human mast cells, which is significantly reduced by both PPADS and NF 449. P2X1 receptor activation, however, does not trigger degranulation. The results indicate that P2X7 receptors may play a significant role in contributing to the unwanted activation of mast cells in chronic inflammatory conditions where extracellular ATP levels are elevated.

**Electronic supplementary material:**

The online version of this article (doi:10.1007/s11302-016-9497-4) contains supplementary material, which is available to authorized users.

## Introduction

Mast cells have long been recognised as key cells involved in immune surveillance. As tissue resident cells, they represent a first line of defence to infection and participate in host protection. In health, the rapid secretion of inflammatory mediators, most notably histamine and proteases, empowers the cells to respond immediately to tissue injury and aid in detoxification, wound healing and tissue remodelling [1]. Consistent with these functions, mast cells are found in tissues which interface the external environment such as skin, airways and gut and are closely associated with blood vessels and sensory nerves. Receptors expressed on their surface enable them to sense the surrounding environment and respond appropriately through the secretion of a wide variety of mediators [2]. However, it has also long been recognised that the inappropriate and/or chronic activation of mast cells is associated with the pathology and symptoms of a wide range of diseases; as the effectors of IgE-mediated type 1 hypersensitivity reactions, mast cells have a well-recognised role in allergic disorders including food allergies, atopic dermatitis, anaphylaxis, rhinitis and asthma. A role for mast cells in other chronic and painful inflammatory conditions including irritable bowel syndrome, progressive kidney disease and migraine is also implicated, although in these cases the triggers involved in mast cell activation are less clear [1, 3–5].

Extracellular ATP is a well-known danger signal associated with inflammatory conditions and has therefore been highlighted as a potential activator of mast cells [6, 7]. In rodent mast cells, ATP, independently of IgE-mediated signalling, acts on P2 purinoceptors [8, 9] of which there are two major groups—the ionotropic P2X receptors and the metabotropic P2Y receptors [10]. P2X receptors are non-selective cation channels which are known to conduct large fractional calcium currents [11] mediating diverse downstream processes such as exocytosis, cytokine release and smooth muscle contraction in a variety of cells [12–14]. It has been shown many times that ATP can induce calcium fluxes in various types of rodent mast cell [15–17], linked to downstream effects such as degranulation [9, 18] and chemotaxis [19]. There are reports that ATP can exert effects on secretion in human mast cells [20, 21], but there are very few specific studies identifying the receptor subtypes involved. The importance of gaining further knowledge with regards the functional role of P2X receptors in human mast cells has recently come to the fore following the discovery that loss-of-function mutations in the P2X7R reduces asthma risk and severity in children [22]; P2X7R on mast cells have also been identified as important contributors to intestinal inflammation in mice and observed to be increased in humans with Crohn’s disease [23]. The potential functional roles of P2X receptors in human mast cells are as yet undetermined, and the involvement of P2X receptors in the ATP-mediated effects on secretion in human mast cells has not been fully explored [7, 24].

We have previously demonstrated the presence of functional P2X1, P2X4 and P2X7 receptors in the LAD 2 human mast cell line and primary human lung mast cells (HLMCs) [25]. The purpose of this study was firstly to determine whether P2X1 and 7 purinoceptors conduct significant calcium influxes in the LAD 2 human mast cell line, and second, to investigate if any calcium fluxes induced via P2X1 and 7 receptor activation can induce human mast cell degranulation.

## Materials and methods

### Human mast cells

LAD 2 mast cells, derived from a patient with mast cell leukaemia, were a gift from Dr. D. Metcalfe (National Institute of Allergy and Infectious Diseases, National Institutes of Health, Bethesda, MD) and cultured in StemPro-34 SFM serum-free complete medium (Invitrogen Life Technologies), with 100 ng/ml SCF as previously described [26]. Half the medium was replaced every 7 days. Cells were maintained at 37 °C in a humidified atmosphere of 5 % CO_2_ incubator.

### Calcium imaging

LAD 2 cells were plated on poly-L-lysine (0.1 %)-coated coverslips, loaded with 1 μM Fura 2-AM and visualised on a Zeiss Axiovert microscope. Cells were perfused with imaging external solution containing (in mM) 142 NaCl, 5 NaHCO_3_, 10 HEPES, 16 glucose, 2 KCl, 2 CaCl_2_, 1 MgCl_2_ and 0.1 % bovine serum albumin (BSA; pH 7.3, NaOH). Images were taken at 1-s intervals at 340 and 380 nm of light (15-ms exposure). The emitted light was passed through a 510–540-nm band pass filter before detection using a cascade 512B CCD camera (Roper Scientific, Photometrics UK). Data was collected and analysed using Metamorph® software (Meta Imaging), and further analysis and graphing were performed using Origin graphing software (OriginPro7.5, Origin corporation, USA). All fluorescence values are background subtracted and displayed as ratio changes. Data represents the mean ± standard error of mean (SEM) unless otherwise stated. Significance was assessed using a Student’s *t* test unless otherwise stated. In all figures, * = *p* < 0.05, ** = *p* < 0.01. Responding cells were counted as those where calcium levels in response to drug rose by more than 5 standard deviations over the baseline in a specified period of time. All concentrations of agonists used were based on previous experiments demonstrating the functional presence of P2X receptors in human mast cells [25].

### β-Hexosaminidase release assays

LAD 2 cells were plated in a 96-well V bottomed plate in imaging buffer and incubated at 37 °C for 10 min. Antagonists were then added as indicated and incubated with the cells at 37 °C for 5–10 min before the addition of agonists. Cells were then incubated at 37 °C for an additional 20 min before being centrifuged (2500RPM, 10 min, 4 °C) and the supernatants removed. Supernatants were incubated with substrate (2 mM 4-nitrophenyl N-acetyl-β-D-glucosaminide diluted in 0.2 M citrate buffer) for 2 h at 37 °C. The reaction was stopped by the addition of Tris-HCl (1 M, pH 9.0) and absorbance at 405 nm measured (Expert Plus Microplate reader, Biochrom Ltd). Spontaneous β-hexosaminidase release was determined by the addition of imaging buffer only. Total β-hexosaminidase content was determined by the addition of Triton X-100 (0.06 %) to lyse the cells. Background readings were determined for later subtraction from wells containing only release buffer and substrate. All test conditions were done in duplicate in a single experiment and each experiment repeated a minimum of three times. Average background values and average spontaneous β-hexosaminidase release values were subtracted from the reading for each well. Release was then expressed as a percentage of the average total β-hexosaminidase content determined from the Triton-treated wells. Results are displayed as mean ± SEM. For all statistical data comparisons, percentage values were log10 converted to transform the data before a one-way ANOVA with post hoc Tukey test was performed. In all figures, * = *p* < 0.05, ** = *p* < 0.01. The LDH cytotoxicity assays were conducted using a kit (Roche) as per the manufacturer’s instructions.

### Reagents

StemPro 34 SFM media and nutrient supplement were obtained from Invitrogen Life Technologies (Paisley, UK). Stem cell factor was obtained from R&D systems (Abingdon, UK). Nucleotides, apyrase, PPADS, poly-L-lysine, EGTA, D-glutamic acid, 4-nitrophenyl N-acetyl-β-D-glucosaminide, Trizma HCl, citric acid and HEPES were purchased from Sigma-Aldrich (Poole, Dorset, UK). Physiological salts, sodium hydroxide and glucose were purchased from BDH anachem. Fura-2 AM was purchased from Calbiochem (Merck Chemicals Ltd., Nottingham, UK) or Invitrogen Ltd. (Paisley, UK). Cesium hydroxide was purchased from ICN Biomedicals Inc. (USA). NF 449 was purchased from Tocris Bioscience (Bristol, UK). AZ 11645373 was a generous gift from AstraZeneca R&D (Charnwood, UK).

## Results

### P2X1 receptor activation induces calcium fluxes in resting LAD 2 cells

**Fig. 1:**
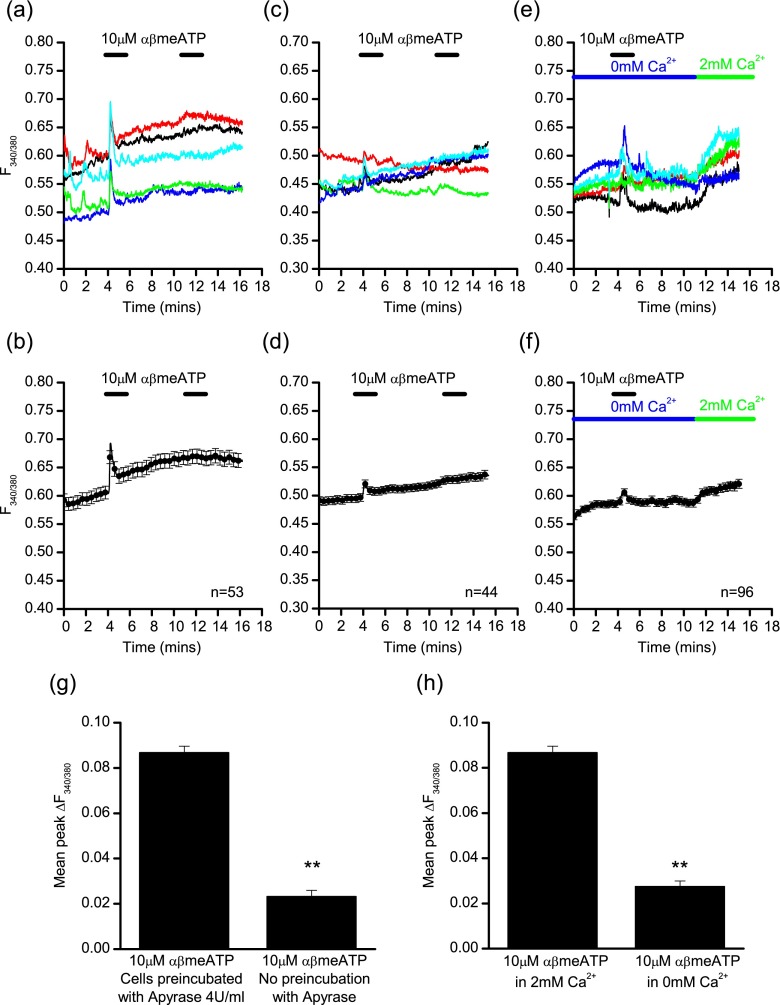
P2X1-mediated calcium fluxes in LAD 2 cells. Example traces (**a**, **c**, **e**), or average responses from one experiment (**b**, **d**, **f**) showing calcium fluxes induced by 10 μM αβmeATP in LAD 2 cells under differing conditions. **a**, **b** following pre-incubation with 4 U/ml apyrase, recorded in the presence of 2 mM calcium; **c**, **d** no pre-incubation with apyrase, recorded in the presence of 2 mM calcium; **e**, **f** following pre-incubation with 4 U/ml apyrase, recorded in calcium free external solution. **g** Summary of the mean peak change in fluorescence ratio induced by 10 μM αβmeATP in LAD 2 cells following pre-incubation or no pre-incubation with 4 U/ml apyrase (independent *t* test, *p* < 0.01, *n* = 126 and *n* = 79 respectively). **h** Summary of the mean peak change in fluorescence ratio induced by 10 μM αβmeATP in LAD 2 cells in the presence or absence of extracellular calcium (independent *t* test, *p* < 0.01, *n* = 126 and *n* = 136 respectively). Data is mean ± SEM and is data from one representative experiment (**b**, **d**, **f**) or pooled data from three separate experiments (**g**, **h**). Drug application is indicated by the *bars* above the traces

To examine whether P2X1 receptors could induce calcium influx in LAD 2 cells, the P2X1 agonist αβmeATP was used. Applying αβmeATP (10 μM) to LAD 2 cells loaded with Fura-2 AM induced a transient increase in intracellular calcium levels (Fig. 1a, b). When cells were pre-incubated with apyrase (4 U/ml, Grade VII) to avoid pre-existing desensitisation of P2X1 receptors, 95 % of cells responded to an initial application of αβmeATP (10 μM) with a mean peak change in fluorescence of 0.087 ± 0.003 a.u. (Fig. 1a, b). This change in fluorescence declined rapidly in the presence of agonist, and a second application failed to elicit a response, consistent with the characteristic desensitisation properties of P2X1 receptors [27, 28]. A significant decrease in the amplitude of the calcium response was observed when cells were not pre-incubated with apyrase (independent *t* test, *p* < 0.01, Fig. 1c, d, g) supporting the presence of pre-existing desensitisation. In all further experiments where P2X1 receptors were studied, cells were pre-incubated with apyrase (4 U/ml, Grade VII, 1 h). When the same experiment was performed in the absence of external calcium (0 mM calcium, 3 mM MgCl_2_, 3 mM EGTA), 59 % of cells responded to an initial application of αβmeATP (10 μM) with a mean peak change in fluorescence of 0.028 ± 0.002 a.u. (Fig. 1e, f). Again, this was a significant reduction in the amplitude of the response when compared to that obtained in the presence of external calcium (independent *t* test, *p* < 0.01, Fig. 1h). This indicates that the predominant pathway for the rise in intracellular calcium is from the external solution, not release from intracellular stores, supporting the involvement of P2X receptors.

**Fig. 2:**
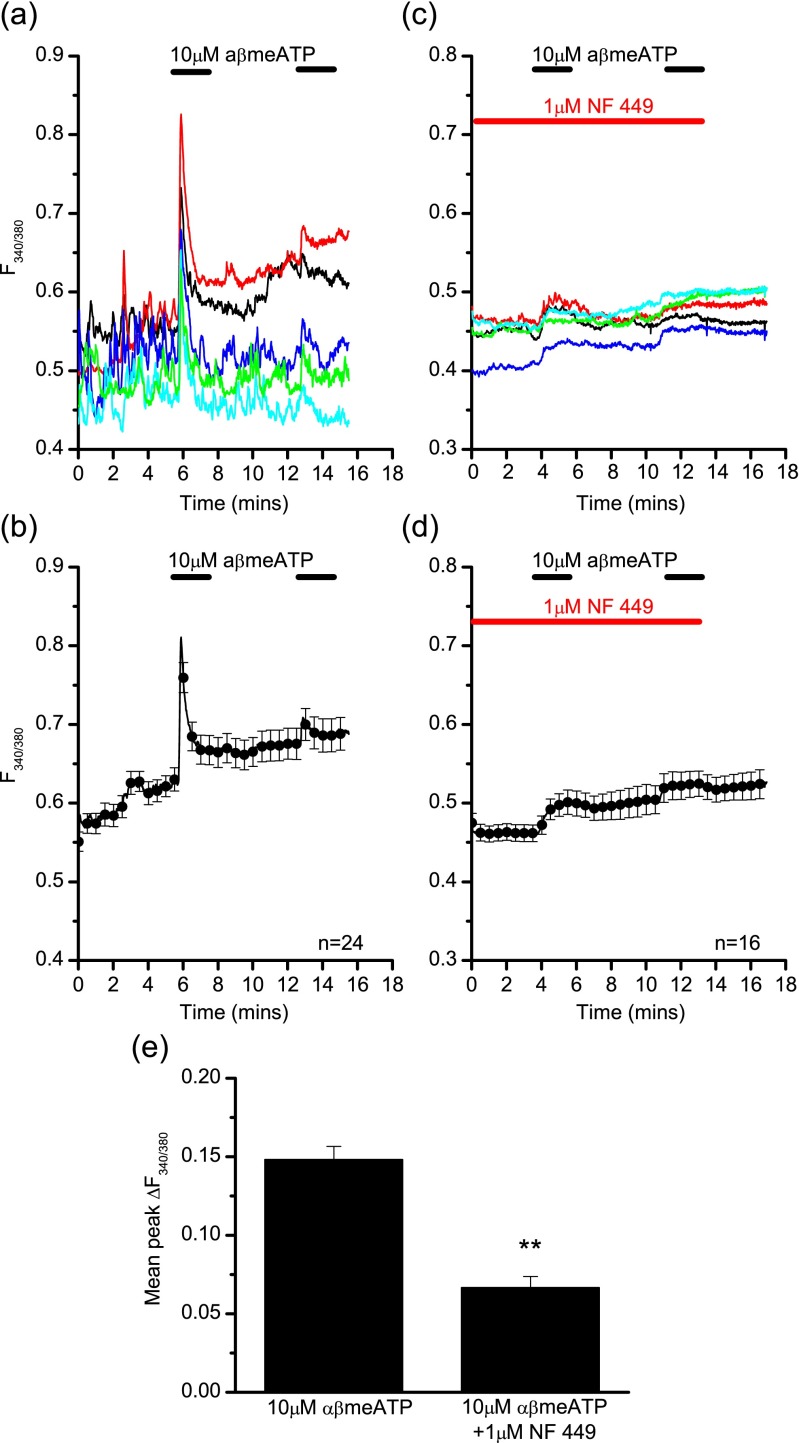
NF 449 antagonism of P2X1-mediated calcium influx in LAD 2 cells. Example traces (**a**, **c**) and average responses from a single experiment (**b**, **d**) showing calcium fluxes in response to 10 μM αβmeATP in LAD 2 cells in the presence (**b**, **d**) or absence (**a**, **c**) of 1 μM NF 449. **e** Summary of the mean peak change in fluorescence in response to 10 μM αβmeATP in LAD 2 cells in the presence, or absence of 1 μM NF 449 (independent *t* test, *p* < 0.01, *n* = 90/82). Data is mean ± SEM and is data from one representative experiment (**b**, **d**) or pooled data from three separate experiments (**e**). All cells were pre-incubated with apyrase (4 U/ml, 1 h). NF 449 was superfused for 5 min prior to agonist and antagonist application. Drug application is indicated by the *bars* above the traces

The calcium influx induced by 10 μM αβmeATP was significantly reduced (independent *t* test, *p* < 0.01, *n* = 59/75) in the presence of the general P2X receptor antagonist PPADS (mean peak ∆F_340/380_ under control conditions was 0.087 ± 0.004 a.u. (*n* = 59 cells, three experiments); mean peak ∆F_340/380_ in the presence of PPADS was 0.027 ± 0.002 a.u. (*n* = 75 cells, three experiments) (data not shown)). The more selective P2X1 antagonist NF 449 (1 μM) also significantly inhibited the αβmeATP (10 μM)-induced calcium influx (independent *t* test, *p* < 0.01), where the mean peak change in fluorescence was reduced from 0.148 ± 0.008 a.u. to 0.067 ± 0.007 a.u. (Fig. 2), supporting P2X1 receptor involvement.

In summary, a transient calcium rise was elicited in LAD 2 cells by the partially selective P2X1 agonist αβmeATP. This rise was reduced in the absence of pre-incubation with apyrase and in the absence of extracellular calcium indicating P2X1 receptor involvement. This calcium flux was sensitive to blockade by both PPADS and NF 449, supporting P2X1 receptor involvement. Taken together, these data indicate that P2X1 receptors stimulate calcium influx in LAD 2 cells under physiological conditions.

### P2X7 receptor activation induces calcium fluxes in resting LAD 2 cells

Previous electrophysiological experiments we have reported demonstrating the functional expression of P2X7 receptors in human mast cells were conducted in low divalent external solution to enhance the magnitude of the signals. Therefore, we first successfully demonstrated the activation of P2X7 receptors under physiological conditions in LAD 2 cells in patch clamp experiments (Supplementary Fig. S1) before measuring calcium fluxes.

**Fig. 3:**
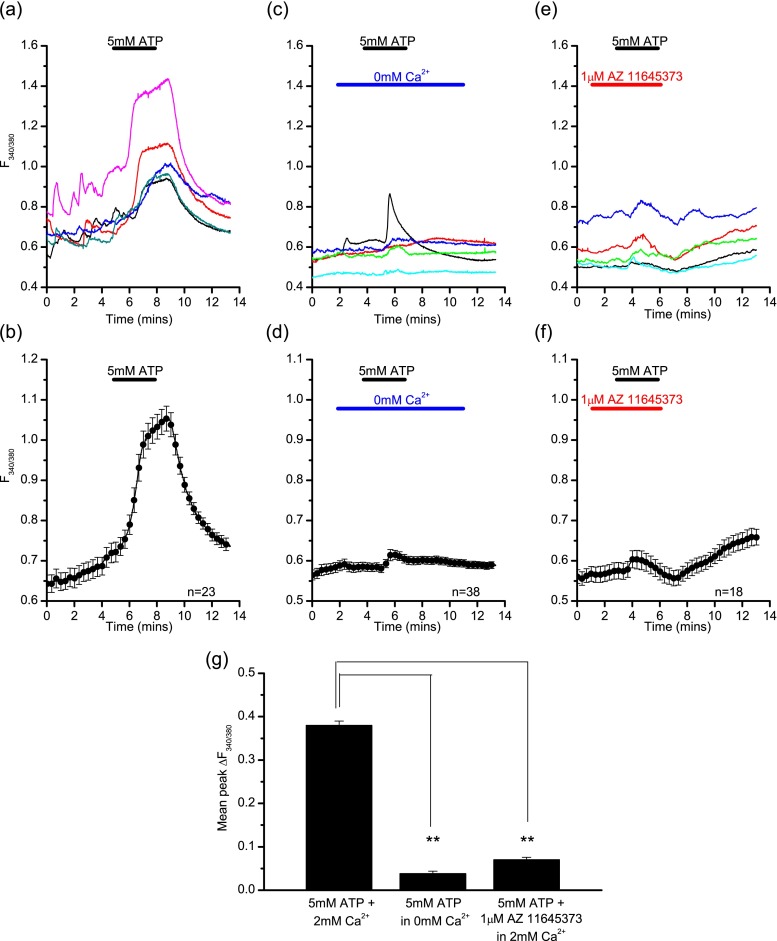
P2X7-mediated calcium fluxes in LAD 2 cells induced by ATP. Example traces (**a**) and the average response (**b**) from a single experiment showing the calcium rise induced by 5 mM ATP in the presence of calcium in LAD 2 cells. Example traces (**c**) and average response (**d**) from a single experiment showing the calcium signal induced by 5 mM ATP in the absence of extracellular calcium in LAD 2 cells. Example traces (**e**) and average response (**f**) from a single experiment showing the calcium signal induced by 5 mM ATP in the presence of AZ 11645373 and extracellular calcium in LAD 2 cells. **g** Summary of the mean peak change in fluorescence induced by 5 mM ATP in the presence or absence of extracellular calcium (independent *t* test, *p* < 0.01, *n* = 137/74) and in the presence of AZ 11645373 (independent *t* test, *p* < 0.01, *n* = 137/58). Data is mean ± SEM and is data from one representative experiment (**b**, **d**, **f**) or pooled data from three to five separate experiments (**g**). Drug application is indicated by the *bars* above the traces

High concentrations of ATP (5 mM) applied to LAD 2 cells loaded with Fura-2 AM induced large calcium influxes (Fig. 3). When stimulated in the presence of extracellular calcium, 99 % of cells responded to 5 mM ATP, with a mean peak change in fluorescence of 0.379 ± 0.011 a.u. The calcium rise began relatively slowly, was non-desensitising and increased during the time of agonist application, corresponding to the characteristics of the current identified previously in patch clamp experiments [25]. Sometimes, a smaller, rapid phase of calcium entry preceded the main calcium rise (seen in Fig. 3a); this was most likely due to the activation of other P2X receptor subtypes (1 and possibly 4) and could also include P2Y-receptor-mediated calcium store release. When the experiment was repeated in the absence of external calcium, 70 % of cells responded to 5 mM ATP with a mean peak change in fluorescence of 0.038 ± 0.006 a.u. (Fig. 3c, d). Under these conditions, the main calcium rise was abolished (independent *t* test, *p* < 0.01, Fig. 3g) with a smaller, rapidly declining calcium signal corresponding to the activation of other receptors as described above. When the cells were perfused with the selective P2X7 antagonist AZ 11645373 (1 μM, 2 min) prior to ATP application, 98 % of cells responded with a mean peak change in fluorescence of 0.071 ± 0.006 a.u. (Fig. 3e, f). Again, the main calcium rise was abolished (independent *t* test, *p* < 0.01, Fig. 3g) leaving a similar small transient calcium signal, like that seen in calcium free conditions. The kinetics of the response to 5 mM ATP, along with the heavy dependence on external calcium levels, indicates P2X7 receptor involvement. This is supported by sensitivity to the selective P2X7 antagonist AZ 11645373.

**Fig. 4:**
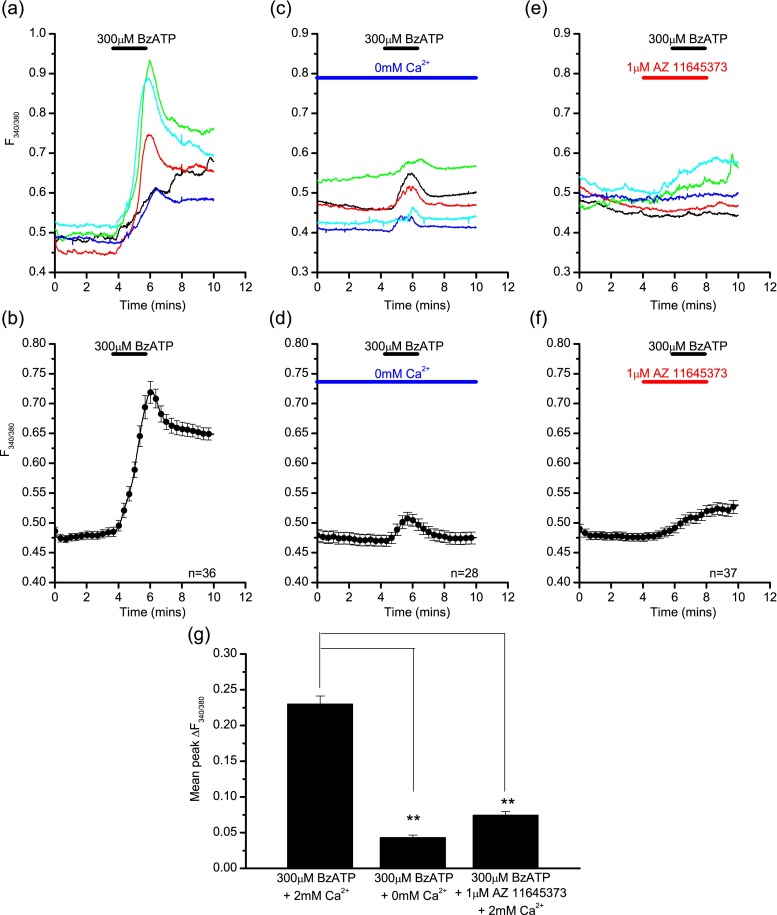
P2X7-mediated calcium fluxes in LAD 2 cells induced by BzATP. Example traces (**a**) and average response (**b**) from a single experiment showing the calcium rise induced by 300 μM BzATP in the presence of extracellular calcium in LAD 2 cells. Example traces (**c**) and average response (**d**) from a single experiment showing the calcium signal induced by 300 μM BzATP in the absence of extracellular calcium in LAD 2 cells. Example traces (**e**) and average response (**f**) from a single experiment showing the calcium signal induced by 300 μM BzATP in the presence of AZ 11645373 and extracellular calcium in LAD 2 cells. **g** Summary of the mean peak change in fluorescence induced by 300 μM BzATP in the presence or absence of extracellular calcium (independent *t* test, *p* < 0.01, *n* = 137/74) and in the presence of AZ 11645373 (independent *t* test, *p* < 0.01, *n* = 137/58). Data is mean ± SEM and is data from one representative experiment (**b**, **d**, **f**) or pooled data from three separate experiments (**g**). Drug application is indicated by the *bars* above the traces

Due to the non-selective actions of ATP, which also potentially activates other P2X and P2Y receptor subtypes, these experiments were repeated using the more selective agonist BzATP. BzATP also activates P2X1 receptors; however, the contribution of these receptors to any response seen is likely to be small and transient, especially as cells were not pre-incubated with apyrase for these experiments. BzATP (300 μM) also elicited a calcium rise in LAD 2 cells (Fig. 4) with similar kinetics to the ATP response, a slowly activating, gradually increasing signal, representing facilitation of the P2X7 receptor. In the presence of extracellular calcium, 98 % of cells responded to BzATP (300 μM) with a mean peak change in fluorescence of 0.229 ± 0.011 a.u. (Fig. 4a, b). This was significantly reduced in the absence of external calcium where 91 % of cells (Fig. 4c, d) responded with a mean peak change in fluorescence of 0.043 ± 0.004 a.u. (independent *t* test, *p* < 0.01, Fig. 4g). As with ATP, the large calcium signal seen in response to BzATP in the presence of calcium was also abolished by the selective P2X7 antagonist AZ 11645373 (mean peak change in fluorescence following AZ 11645373 treatment of 0.074 ± 0.005 a.u.; independent *t* test, *p* < 0.01, Fig. 4e, f, g), further supporting P2X7 receptor involvement in this response.

**Fig. 5:**
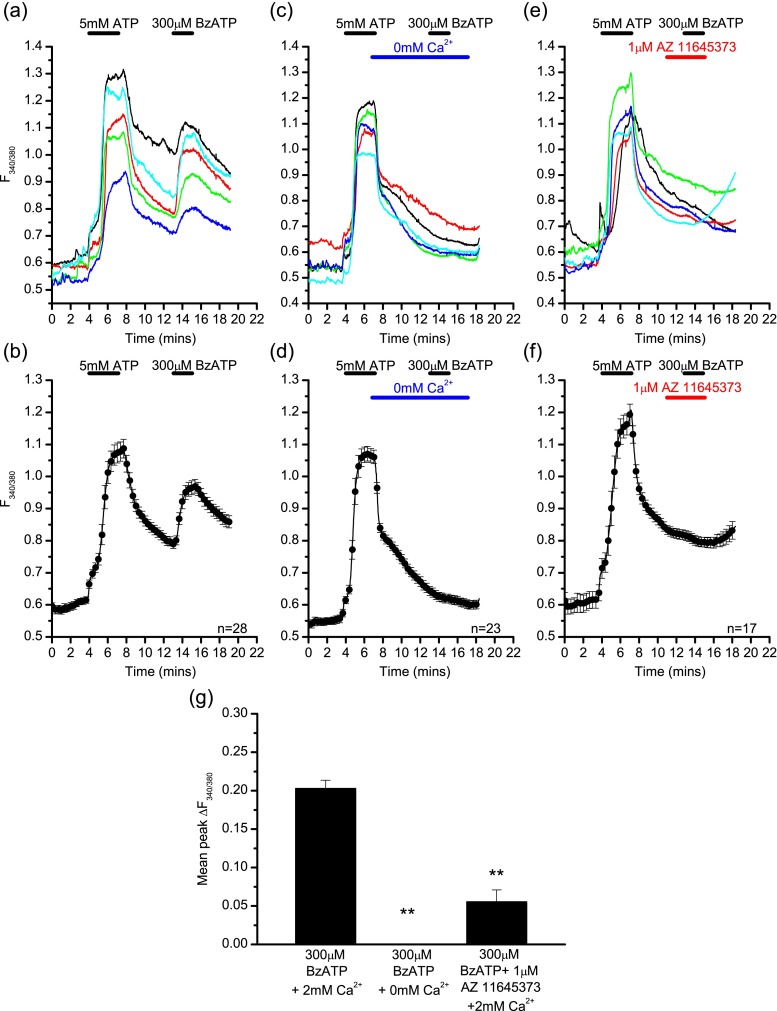
BzATP-mediated calcium fluxes in LAD 2 cells following ATP application. Example traces (**a**) and average response (**b**) from a single experiment showing the calcium rise in response to 5 mM ATP followed by 300 μM BzATP in the presence of extracellular calcium. Example traces (**c**) and average response (**d**) from a single experiment showing the calcium rise in response to 5 mM ATP followed by 300 μM BzATP in the absence of extracellular calcium. Example traces (**e**) and average response (**f**) from a single experiment showing the calcium rise in response to 5 mM ATP followed by 300 μM BzATP in the presence of AZ 11645373. **g** Summary of the mean peak change in fluorescence induced by 300 μM BzATP following a 5-mM ATP application in the presence or absence of extracellular calcium (independent *t* test, *p* < 0.01, *n* = 137/78), or in the presence of absence of AZ 11645373 (independent *t* test, *p* < 0.01, *n* = 137/49). Data is mean ± SEM and is data from one representative experiment (**b**, **d**, **f**) or pooled data from three separate experiments (**g**). Drug application indicated by the bars above the traces

When applying BzATP (300 μM) after a facilitating application of ATP (5 mM), a second calcium rise with similar kinetics was seen (Fig. 5a, b). The initial ATP application would be expected to desensitise other P2X and P2Y receptors before the application of BzATP, thereby further isolating the P2X7-mediated calcium rise. When applied in the presence of calcium, the mean peak change in fluorescence in response to 300 μM BzATP was 0.203 ± 0.011 a.u. This response was abolished in the absence of calcium (mean peak change in fluorescence of −0.031 ± 0.002 a.u.; independent *t* test, *p* < 0.01, Fig. 5c, d), and in the presence of AZ 11645373 (mean peak change in fluorescence of 0.055 ± 0.015 a.u.; independent *t* test, *p* < 0.01, Fig. 5e, f), further supporting evidence that the calcium rise was mediated by P2X7 receptors.

The finding that high concentrations of both ATP and BzATP bring about calcium rises in LAD 2 cells which are dependent on extracellular calcium and are sensitive to the P2X7 selective antagonist AZ 11645373 indicate that these responses are mediated to a large extent by P2X7 receptors.

### ATP-induced mediator release in LAD 2 cells

**Fig. 6:**
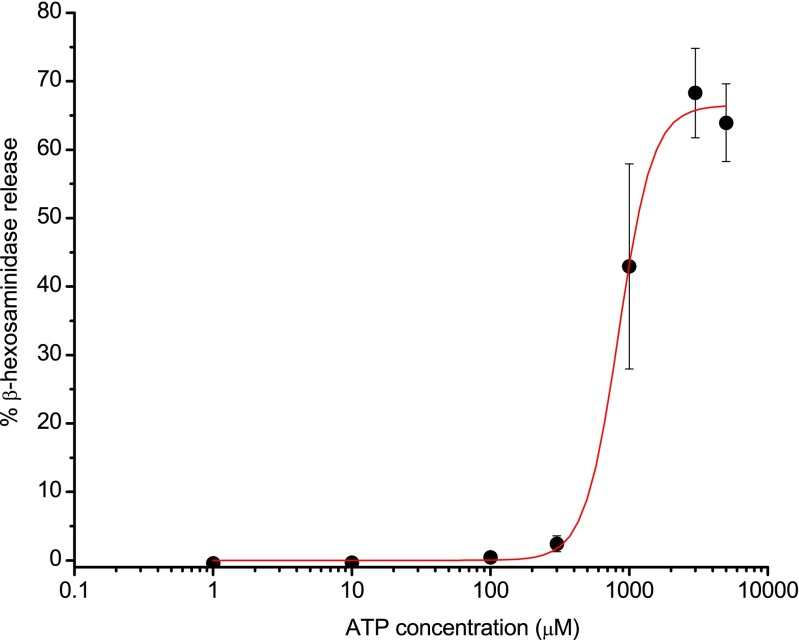
β-Hexosaminidase release in response to ATP in LAD 2 cells. Concentration response curve to ATP showing β-hexosaminidase release. Data fit with the Hill equation (*y* = *V*_max_*x ^*n*^ / (*K* ^*n*^ + *x* ^*n*^), using Origin v7.5 software. Fit shown in *red*, no weighting applied. EC_50_ value of mean data = 0.84 mM ATP. Hill slope = 3.5. Data is mean ± SEM, *n* = 4 experiments performed in duplicate or triplicate

β-Hexosaminidase is a granule-associated mediator known to be released by LAD 2 cells upon stimulation with antigen [29]. To examine the potential role of P2X receptors in human mast cell mediator release, a concentration response curve for β-hexosaminidase release from LAD 2 cells in response to ATP was constructed (Fig. 6). There was no detectable release at low concentrations of ATP (1 and 10 μM), indicating a lack of P2X1-mediated secretion. Raising the concentration to 100 and 300 μM caused very low levels of release (0.5 ± 0.5 and 2.4 ± 1.2 %, respectively). At high concentrations of ATP (1, 3 and 5 mM), β-hexosaminidase release was markedly increased, appearing to plateau at 5 mM ATP with release of 63.9 ± 5.7 %; this coincides with the concentrations of ATP that would activate P2X7 receptors. The concentration response mean data was fitted using the Hill equation, which yielded an EC_50_ value of 0.84 mM ATP.

Due to the fact that prolonged exposure to ATP can lead to apoptosis in P2X7-expressing cells [30, 31], it was important to establish that the β-hexosaminidase in the supernatant at high concentrations of ATP was not due to cell lysis and death. Cell counts and Trypan blue dye exclusion indicated that the overall percentage of dead cells following a release assay was similar when they were stimulated with either 1 μM (4.6 %) or 5 mM (6.3 %) ATP (Table 1). This indicates that the β-hexosaminidase in the supernatant was present due to receptor stimulation and not non-specific cell death or apoptosis.

**Table 1:** Cell viability following P2X7 receptor activation in β-hexosaminidase release assays

Experiment	% cells dead following release assay			
	1 μM ATP	5 mM ATP	1 μM BzATP	300 μM BzATP
1	3.6	9.5	4.5	3.9
2	4.5	3.5	7.7	5.0
3	2.9	2.6	1.3	5.8
4	7.5	9.7		
Average	4.6	6.3	4.5	4.9

Table shows the percentage of dead cells present following release assays; cell viability was determined by Trypan blue dye exclusion (cells stained with 0.05 % Trypan blue dye in PBS); *n* = 3/4 experiments

The protocol for these release assays involved incubating the cells with agonist for 20 min. Unlike imaging experiments, where the solution bathing the cells was continually refreshed, in this experiment the cells were in contact with the same solution for the whole time period. This is important as mast cells have been reported to express ectonucleotidases [32, 33] which can degrade ATP to ADP, AMP and adenosine, all of which could secondarily activate mast cells via receptors other than P2X and ATP sensitive P2Y receptors. It is therefore important to examine each of the receptor subtypes in isolation with more stable and selective agonists.

### Lack of P2X1-mediated β-hexosaminidase release in LAD 2 cells

**Fig. 7:**
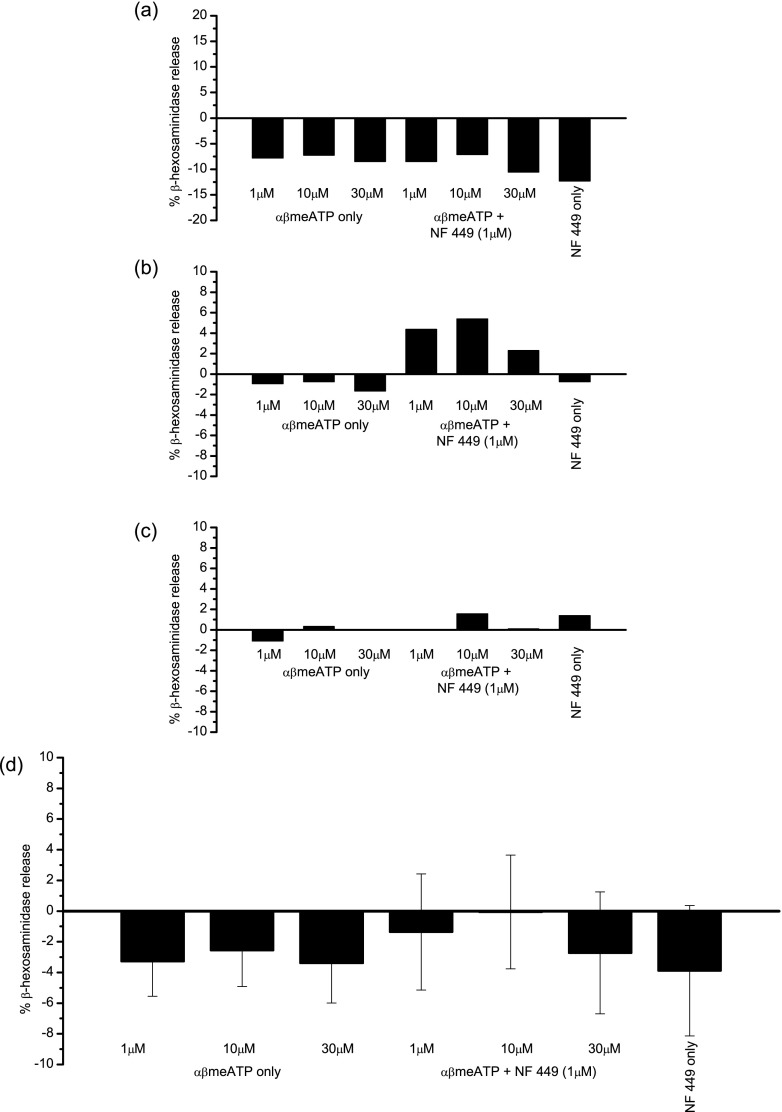
P2X1 receptor activation and β-hexosaminidase release in LAD 2 cells. **a**, **b**, **c** Examples of individual β-hexosaminidase release assays (average of duplicate wells) in response to αβmeATP (1, 10 or 30 μM) with or without NF 449 (1 μM). **d** Summary of mean β-hexosaminidase release in response to αβmeATP (1, 10 or 30 μM) with or without NF 449 (1 μM). Cells were pre-incubated with apyrase (Grade VII, 4U/ml, 1 h). Data is mean ± SEM, *n* = 3 experiments performed in duplicate. One-way ANOVA, *p* > 0.05

To specifically address the role of P2X1 receptors in human mast cell secretion, LAD 2 cells were stimulated with αβmeATP (1, 10 and 30 μM) either with or without the antagonist NF 449 (1 μM) following pre-incubation with apyrase (Grade VII, 4U/ml, 1 h). These compounds induced no discernable pattern of stimulation or inhibition of release (Fig. 7, one-way ANOVA, *p* > 0.05).

### P2X7-mediated β-hexosaminidase release in LAD 2 cells

**Fig. 8:**
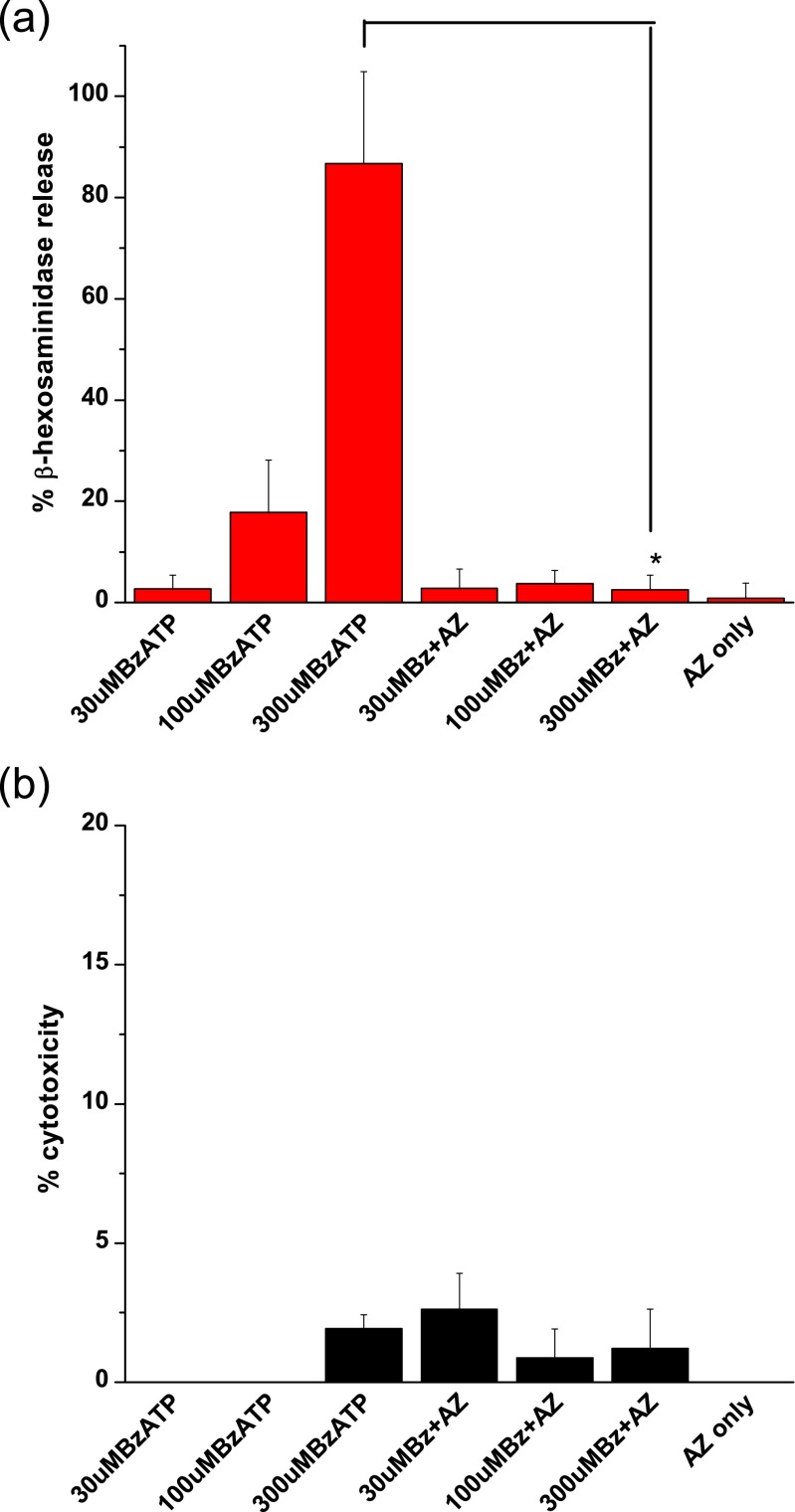
β-Hexosaminidase release in response to BzATP in LAD 2 cells. **a** Dose-dependent release of β-hexosaminidase from LAD 2 cells in response to BzATP which can be inhibited by AZ 11645373 (one-way ANOVA, post hoc Tukey test, *p* < 0.05, *N* = 4). **b** Lack of BzATP induced cytotoxicity in LAD 2 cells as measured by LDH release

When stimulated with the more selective agonist BzATP, a dose-dependent release of β-hexosaminidase was again seen with striking release at 300 μM BzATP (88.8 ± 12.5 %, Fig. 8a). This release was completely abolished in the presence of AZ 11645373 (3.4 ± 1.6 %, one-way ANOVA, post hoc Tukey test, *p* < 0.05). This further suggests the involvement of P2X7 receptors in this mediator release. Again, cell viability counts revealed a similar percentage of dead cells following the release assay in wells stimulated with 1 μM BzATP (4.5 %) and 300 μM BzATP (4.9 %; Table 1). LDH measurements indicated a lack of significant cytotoxicity (300 μM BzATP associated with 1.6 % ± 0.5 % cytotoxicity, Fig. 8b), supporting the hypothesis that the β-hexosaminidase was actively secreted following stimulation of P2X7 receptors rather than leaking from dead and dying cells. A lack of external calcium also abolished release indicating a physiological process (data not shown).

## Discussion

This study aimed to determine whether P2X receptors could induce calcium fluxes and degranulation in human mast cells. Single-cell imaging experiments revealed that calcium flux through P2X1 receptors is fast and transient, augmented by pre-incubation with apyrase, desensitises upon repeated agonist application and is antagonised by PPADS and NF 449. The effects of apyrase on αβmeATP-induced calcium signals are consistent with the view that P2X1 receptors may become desensitised by the presence of ATP in the growth media, an effect which may obscure the function of these receptors in in vitro studies [34, 35]. P2X7-mediated calcium rises in human mast cells by contrast are sustained and facilitate over the duration of agonist application and are antagonised by AZ 11645373. In the absence of extracellular calcium, a small calcium signal could still be seen to both αβmeATP and ATP in LAD 2 cells, suggesting that purinergic receptors can also induce store-dependent calcium signals in these cells. Moreover, as a residual calcium signal could also be seen following antagonism of the P2X responses, it is likely that these signals arise from P2Y receptors, all of which are reported to be expressed in LAD 2 cells [36]. It therefore seems likely that in native systems, complex calcium signals incorporating influx through P2X1 receptors, together with store-released calcium signalling arising from activation of phospholipase C coupled P2Y receptors, are likely to be generated in mast cells exposed to micromolar levels of ATP. On the other hand, when ATP levels are increased to millimolar concentrations, for example in pathological conditions, signals generated by P2X1 receptors, and possibly P2Y receptors, are likely to be reduced as a result of desensitisation and superceded by calcium influx through P2X7 receptors. The differences in the amplitude and kinetics of the calcium signals generated by different levels of ATP, and P2X1 versus P2X7 receptors, are likely to have a significant impact on the downstream functions each regulates in human mast cells [37].

Due to the importance of mediator release in mediating mast cell function and pathology [2], and the prominent role calcium signalling plays in regulating secretion [38], the downstream consequences of P2X receptor activation on mast cell degranulation were evaluated. The main components of mast cell granules include proteases such as tryptase, chymase, carboxypeptidase and histamine. The lysosomal enzyme, β-hexosaminidase, is also stored and released from mast cell granules and is therefore frequently used as a way to measure mast cell degranulation in vitro [39]. Here, we show that under conditions in which P2X1 receptors support calcium influx across the plasma membrane, no β-hexosaminidase is released. Notably, a previous study in LAD 2 mast cells reported that activation of P2Y receptors also does not induce degranulation [36], supporting the notion that in vivo, mast cells do not degranulate in response to low levels of ATP. In contrast, at high concentrations of ATP, and in response to BzATP, significant release of β-hexosaminidase was observed. Moreover, the release of β-hexosaminidase following P2X7 activation was dependent upon extracellular calcium, indicating it resulted from regulated exocytosis of β-hexosaminidase rather than leakage from dead or dying cells. The lack of cytotoxicity to high concentrations of BzATP was confirmed with an LDH assay. The sensitivity of this β-hexosaminidase release to the selective P2X7 antagonist AZ 11645373 is supporting evidence that the release can be attributed to P2X7 receptors.

The results from this and other studies add to the growing list of receptors identified on mast cells able to induce IgE-independent activation [2], and show that degranulation of mast cells is not solely regulated by calcium influx through Orai channels [37], but may also couple to P2X7 receptors. Already, a host of studies have reported ATP-induced mediator release in rodent mast cells, ranging from calcium-dependent histamine release, to an upregulation in transcription and secretion of pro-inflammatory cytokines [7]. In human mast cells, most studies to date have demonstrated modification of IgE-stimulated mediator release but not release induced by ATP alone. One study using human cord blood derived mast cells found that ATP or ADP alone did not induce β-hexosaminidase release but together were a weak stimulus for release; in addition, they both modified IgE-dependent secretion [21]. Similar results were obtained from HLMCs, where a potentiation of IgE-mediated secretion by ATP was attributed to P2Y1 and P2Y2 receptors [20]. The potential for P2X receptors to modify other mast cell functions, including the cocktail of mediators secreted under different stimulus conditions, warrants further investigation. In addition to regulated exocytosis of preformed mediators, “activated” mast cells synthesise lipid mediators, cytokines, chemokines and growth factors which can be released independently or in consort with preformed mediators. Calcium plays a key role in regulating the synthesis of most of these mediators; it is possible and probable that calcium signals generated by the P2X receptor subtypes identified in human mast cells can also influence eicosanoid and cytokine/chemokine synthesis and thereby influence mast cell functions in health and disease.

The release of pro-inflammatory mediators is a key part of the development of the symptoms of allergic disease [40], and identifying any receptors or ion channels contributing to this could lead to novel therapeutic targets. Through their participation in type 1 hypersensitivity reactions, mast cells are involved in a range of allergic disorders including atopic dermatitis, anaphylaxis and asthma. Following an initial allergic reaction, for example in the lung of an asthmatic, an acute inflammatory reaction occurs in the surrounding tissues [41]. As this response takes effect, ATP levels would be expected to rise in the extracellular environment, possibly contributed to by release from mast cells themselves [42]. In support of this, elevated levels of ATP and its breakdown product adenosine have been found in BAL fluid of asthmatics [43, 44]. This provides a pathway for the activation of P2Y, P2X and adenosine receptors on multiple cell types within the lung, including mast cells, and the importance of ATP and adenosine in asthma has been demonstrated by the finding that they can both induce bronchoconstriction in asthmatic patients [45, 46]. Recent reports on the lower asthma rates in children with attenuated P2X7 function further support a role for P2X7 receptors in the process and their importance as a therapeutic target [22].

We have previously demonstrated the presence of functional P2X7 receptors in human lung mast cells [25]; given the recent corroborating evidence for the involvement of P2X7 receptors in asthma risk and severity in children, and in another chronic inflammatory condition involving mast cells, namely Crohn’s disease, the results of our study strongly support the potential repositioning of P2X7 selective antagonists for the treatment of chronic inflammatory conditions in which mast cells play a significant role.

## Electronic supplementary material

Below is the link to the electronic supplementary material.

Supplementary Fig. 1 Concentration response curve of P2X7 receptors to ATP under physiological conditions in LAD 2 cells. (a) Superimposed traces (labelled 1-7) from a single cell showing facilitation of P2X7-like responses to repeated applications of ATP (5 mM, 1 minute intervals) in ‘normal’ recording solution, i.e. not low divalent. (b) Example trace of the protocol used to ensure full facilitation of P2X7-like responses before starting the dose response curve. 5 mM ATP was applied continuously for 30s (left panel), then at 1 minute intervals for 10s until a reproducible, plateau response was obtained (right panel). (c) Superimposed traces from a single cell in response to differing concentrations of ATP. (d) P2X7-like receptor concentration response curve to ATP shown as mean peak currents normalised to the maximal 10 mM ATP response. Data is mean +/- SEM, n=5. Data fit with the Hill equation (y=Vmax*x^n^/(K^n^+x^n^), using Origin v7.5 software. Fit shown in red, no weighting applied. EC_50_ value of the mean data = 3.4 mM ATP. Whole-cell patch clamp recordings performed at room temperature, using an EPC10 amplifier and Pulse acquisition software (HEKA, Lambrecht, Germany). Membrane clamped at -60 mV. External solution contained in mM: 147 NaCl, 10 HEPES, 16 Glucose, 2 KCl, 2 CaCl_2_, and 1 MgCl_2_ (pH 7.3, NaOH). Internal solution contained in mM: 135 D Glutamic acid, 8 NaCl, 10 EGTA, 10 HEPES, 3.6 CaCl_2_ and 2 MgATP (pH 7.3, CsOH), omitting MgATP from the tip. (GIF 14 kb)

High Resolution Image (EPS 8780 kb)

## Abbreviations

ATP: Adenosine 5′-triphosphate
αβmeATP: Alpha beta methyl ATP
BzATP: 2′(3′)-*O*-(4-Benzoylbenzoyl)-ATP
NF 449: 4,4′,4″,4″′-[Carbonylbis(imino-5,1,3-benzenetriyl-b*is*(carbonylimino))]*tetrakis*-1,3-benzenedisulfonic acid, octasodium salt
PPADS: Pyridoxal-5-phosphate-6-azophenyl-2′,4′-disulphonic acid
