# Supplementary figures and images for: P2X7 receptors induce degranulation in human mast cells
Source: Purinergic Signal. 2016 Feb 24;12(2):235–46. doi: 10.1007/s11302-016-9497-4 (PMC4854833; doi:10.1007/s11302-016-9497-4)

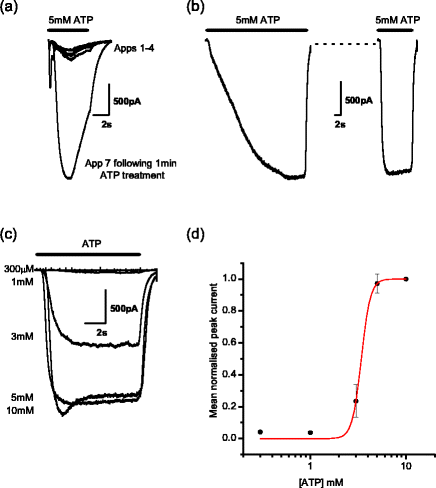

Supplement: Supplementary file 1 — Concentration response curve of P2X7 receptors to ATP under physiological conditions in LAD 2 cells. (a) Superimposed traces (labelled 1-7) from a single cell showing facilitation of P2X7-like responses to repeated applications of ATP (5 mM, 1 minute intervals) in ‘normal’ recording solution, i.e. not low divalent. (b) Example trace of the protocol used to ensure full facilitation of P2X7-like responses before starting the dose response curve. 5 mM ATP was applied continuously for 30s (left panel), then at 1 minute intervals for 10s until a reproducible, plateau response was obtained (right panel). (c) Superimposed traces from a single cell in response to differing concentrations of ATP. (d) P2X7-like receptor concentration response curve to ATP shown as mean peak currents normalised to the maximal 10 mM ATP response. Data is mean +/- SEM, n=5. Data fit with the Hill equation (y=Vmax*xn/(Kn+xn), using Origin v7.5 software. Fit shown in red, no weighting applied. EC50 value of the mean data = 3.4 mM ATP. Whole-cell patch clamp recordings performed at room temperature, using an EPC10 amplifier and Pulse acquisition software (HEKA, Lambrecht, Germany). Membrane clamped at -60 mV. External solution contained in mM: 147 NaCl, 10 HEPES, 16 Glucose, 2 KCl, 2 CaCl2, and 1 MgCl2 (pH 7.3, NaOH). Internal solution contained in mM: 135 D Glutamic acid, 8 NaCl, 10 EGTA, 10 HEPES, 3.6 CaCl2 and 2 MgATP (pH 7.3, CsOH), omitting MgATP from the tip. (GIF 14 kb) [file 11302_2016_9497_Fig9_ESM.gif]
